# Pro-osteogenic effects of fibrin glue in treatment of avascular necrosis of the femoral head *in vivo* by hepatocyte growth factor-transgenic mesenchymal stem cells

**DOI:** 10.1186/1479-5876-12-114

**Published:** 2014-05-07

**Authors:** Qian Wen, Chaoying Zhou, Wei Luo, Mingqian Zhou, Li Ma

**Affiliations:** 1 Institute of Molecular Immunology, School of Biotechnology, Southern Medical University, #1838, Northern Guangzhou Ave, Guangzhou, Guangdong 510515, People’s Republic of China

**Keywords:** Avascular necrosis of the femoral head, Mesenchymal stem cell, Fibrin glue, Proliferation, Differentiation, Osteogenic regeneration

## Abstract

**Background:**

Autologous transplantation of modified mesenchymal stem cells (MSCs) is a promising candidate for the treatment of the refractory clinical disease, avascular necrosis of the femoral head (ANFH). Our previous attempts by compounding MSCs with medical fibrin glue to treat ANFH in animal model have achieved excellent effects. However, the underlying molecular mechanism is unclear, especially on the transgenic gene expression.

**Methods:**

Rabbit MSCs were isolated and compounded with fibrin glue. Following degrading of fibrin glue, proliferation, viability, expression of transgenic hepatocyte growth factor gene as well as osteogenic differentiation of MSCs were evaluated together with that of uncompounded MSCs. Fibrin glue-compounded MSCs were transplanted into the lesion of ANFH model, and the therapeutic efficacy was compared with uncompounded MSCs. One-Way ANOVA was used to determine the statistical significance among treatment groups.

**Results:**

Fibrin glue compounding will not affect molecular activities of MSCs, including hepatocyte growth factor (HGF) secretion, cell proliferation and viability, and osteogenic differentiation *in vitro*. When applying fibrin glue-compounded MSCs for the therapy of ANFH *in vivo*, fibrin glue functioned as a drug delivery system and provided a sustaining microenvironment for MSCs which helped the relatively long-term secretion of HGF in the femoral head lesion and resulted in improved therapeutic efficacy when compared with uncompounded MSCs as indicated by hematoxylin-eosin staining and immunohistochemistry of osteocalcin, CD105 and HGF.

**Conclusion:**

Transplantation of fibrin glue-compounding MSCs is a promising novel method for ANFH therapy.

## Background

As an irreversibly progressive pathological process, avascular necrosis of the femoral head (ANFH) is regarded as an immortal and incurable disease which mainly afflicts people under 40-year-old [1-3]. Mesenchymal stem cells (MSCs)-based treatments are believed to be a promising method for ANFH therapy due to the osteogenic potential of MSCs and the convenience of preparation [4], and have been applied for therapy of ischemic diseases such as ANFH [5-7] and myocardial infarction [8,9] with satisfactory effects.

Medical fibrin glue is a biopolymer matrix composed by human natural fibrinogen and thrombin. Fibrin glue is commonly used for surgical hemostasis and tissue sealing as a sealant [10-12]. In recent years it has been applied as supporting material for cell growth, migration, and differentiation [13,14]. Fibrin glue can also be used as the cell delivery system in tissue engineering [15-17], such as construction of an injectable tissue-engineered bone graft, or as the drug delivery system in various drug therapies [18-20]. The advantages of clinical application of fibrin glue were widely studied, yet the detailed underlying molecular mechanism still need further exploration, such as long-term sustainable effects in MSCs-medicated treatments.

We previously reported the significant effects of adenoviral vector-based hepatocyte growth factor (Ad-HGF)-transfected MSC treatments using the model of hormone ANFH in which MSCs have ever been compounded with a supportive fibrin glue delivery mixture and applied in the local ANFH lesion region [21]. However, in a short-term observation period of 4 weeks post treatment, there was no significant difference in the recovery efficacy regardless of whether using compounded or uncompounded MSCs, and also the effects of fibrin glue on MSCs activity was not evaluated.

In this work, we systemically compared the therapeutic efficacy of HGF-transgenic MSCs by compounded or uncompounded with fibrin glue and extended the observation time to as long as 8 weeks following treatment. Here, we showed the evidences of the molecular effects and mechanism of fibrin glue-compounded MSCs in improving the effects of ANFH therapy, which may improve our understanding of the role of fibrin glue in MSC treatment of ANFH and shade light on the generation of better therapeutic regime.

## Materials and methods

### Preparation and differentiation of BMSCs

Allogeneic MSCs were prepared, identified and transfected with Adenovirus vectors as described before [21]. For identification and differentiation assays of BMSCs, cells were induced to differentiate into osteoblasts, chondroblasts and adipocytes separately, by culturing cells in corresponding induction media (Cyagen Biosciences Inc., Goleta, CA). The differentiation characteristics were detected using NBT-BCIP Alkaline Phosphatase Color Development Kit (Beyotime Institute of Biotechnology, Shanghai, China) and Alizarin Red Sulfate (ARS) staining (Cyagen) for osteogenic differentiation, Alcian blue staining (Cyagen) for chondrogenic differentiation and Oil Red O staining (Cyagen) for adipogenic differentiation according to the instruction of the manufactures.

### Preparation of transfected BMSCs compounding with medical fibrin glue

For compounding with fibrin glue, Ad-HGF or Ad-GFP-transfected MSCs were collected 48 h later. Two component solutions of fibrin glue were prepared separately. In solution I, freeze-dried human fibrinogen (Shanghai RAAS Blood Products Co. Ltd., Shanghai, China) was dissolved to 80 mg/mL with 2000 KIU/mL aprotinin in sterile water (Lanzhou Dadeli Biochemical Pharmaceutical Co. Ltd., Lanzhou, China) at 37°C. In solution II, thrombin (Jin Kang Pharmaceutical Co. Ltd., Nanjing, China) was dissolved to 400 IU/mL with 40 mmol/L of CaCl_2_ in sterile water. For *in vitro* assays, 2 × 10^4^ cell pellet was first resuspended with 180 μL of solution I, and quickly mixed with 20 μL of solution II in one well of 24-well plates (Nunc, Thermo Fisher Scientific, Waltham, MA). The MSC-fibrin glue composite was cultured and observed for 1 week. For ANFH therapy *in vivo*, a duplex syringe was used to transfer 90 μL of solution I containing 1 × 10^6^ transfected MSCs and 10 μL of solution II into the lesions through the tunnel of the core decompression as described previously [21].

### Scanning electron microscopy (SEM) sample preparation and observation

For SEM observation, fibrin glue composites containing MSCs were cut into small pieces and fixed with pre-cold fresh 2.5% glutaraldehyde-0.1 M PBS (pH7.4) at 4°C for 1 h. Following wash 3 times with PBS, samples were post-fixed with 1% osmium tetroxide-0.1 M PBS for 30 min and stained with 1% tannic acid-0.1 M PBS for 30 min. After dehydration through gradient concentration of ethanol, the specimens were dried at critical point and coated with gold by ion sputter, and observed with S-3000 N scanning electron microscope (Hitachi, Hitachi, JP).

### HGF ELISA

To assay HGF secretion by MSCs compounded with fibrin glue, one week after compounding, fibrin glue was dissolved using 1600 U/mg of bovine pancreas trypsin (Calbiochem, La Jolla, CA) at 37°C. Cells released from fibrin glue composite were cultured for 24 h, and the HGF secretion in the supernatant was analyzed using an ELISA kit (BioSource International, Inc., Camarillo, CA) as per the manufacturer’s instruction. Results were read at 450 nm wavelength using a Varioskan Flash microplate reader (Thermo Fisher Scientific).

### Cell proliferation and viability assays

The proliferation activity and viability of MSCs compounding with or without fibrin glue were assayed using the Cell-Light™ EdU DNA Cell Proliferation Kit (Guangzhou Ribobio Co., Ltd, Guangzhou, China) and the Cell Counting Kit-8 (CCK-8, Dojindo Laboratorise, Tokyo, Japan) according to the manufacturer’s instruction and as described before [22].

### Animal surgery and MSC injection

Thirty New Zealand rabbits were maintained under specific pathogen-free conditions in The Experimental Animal Center of Nanfang Hospital (Guangzhou, China) and randomly assigned to 4 groups with the approve of the Animal Ethics Committee at Southern Medical University. Normal group included 5 animals untreated, while horse serum (Hyclone) and prednisolone acetate (Pharmacia & Upjohn Co., Kalamazoo, MI) were used to induce early stage of hormone ANFH in the remaining 25 animals as previously described [23]. Model group contained 5 rabbits with untreated ANFH. The remaining animals were separated evenly to 2 groups to receive treatment of HGF-transgenic MSC transplantation compounded with fibrin glue (HGF/MSCs + FG) or uncompounded (HGF/MSCs) as described previously [22]. To further demonstrate the activities of HGF-transgenic MSCs, the efficacy was further compared with another four treatments: FG only (FG), HGF-transgenic fibroblasts compounded with fibrin glue (HGF/Fibroblasts + FG), GFP-transgenic MSCs compounded with fibrin glue (GFP/MSCs + FG) and HGF-transgenic osteoblasts compounded with fibrin glue (HGF/OB + FG). Animals all survived during the observation periods and femoral head samples were obtained following air-euthanasia at 4 and 8 weeks post-treatment. Paraffin embedded-samples and sections were assayed with hematoxylin-eosin (HE) staining and immunohistochemistry with the following antibodies: osteocalcin (OCN) (OCG4; Abcam plc., Cambridge, UK; 1:100), CD105 (SN6h; Invitrogen co. Ltd., CA, USA; 1:75), and HGF (BOSTER Bioengineering Co. Ltd., Wuhan, China; 1:250), p-ERK1/2 (E-4; 1:250), p-Akt (D9E; Santa Cruz Biotechnology, Inc., Santa Cruz, CA, USA; 1:250). Pictures were taken with microscopy (Nikon), while the number of empty lacunae and hematopoietic medullary cells as well as the integrated optical density (IOD) in 10 random fields per section was analyzed with Image-Pro Plus software version 6.0 (IPP 6.0, Media Cybernetics, Inc., MD). Three sections per animal were analyzed. Calculation of IOD was carried out according to the instruction of the software. After lining out positive staining areas in the sections, the area and the color depth were synthetically calculated by the software.

### Statistical analysis

Data were expressed as the mean ± se. Statistical significance was set at *P* < 0.05 determined by One-Way ANOVA using SPSS statistical software version 16.0 (SPSS, Chicago, IL). Post hoc multiple comparisons were done with least Significant Difference or Dunnett’s T3.

## Results

### Characterization of MSCs and morphology in compounded fibrin glue

**Figure 1:**
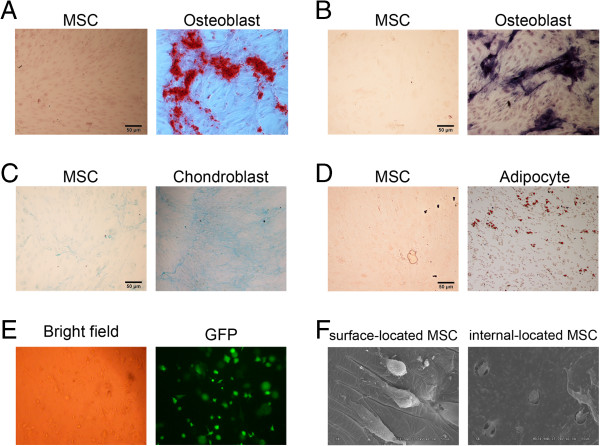
**Identification of multi-potential differentiation of MSCs and observation of MSCs compounded with fibrin glue.** Cells were cultured and induced to differentiate into osteoblasts indicated by staining of Alizarin Red **(A)** and NBT-BCIP **(B)**, chondroblasts by Alcian blue **(C)** and adipocytes by Oil Red O **(D)**. **(E)** After fibrin glue-compounding within one week, normal cell shape under bright field and green fluorescence resulting from Ad-HGF transfection indicated the cell activity. **(F)** The morphologies of cells on the surface of and inside the fibrin glue were observed with the scanning electric microscopy.

First, we isolated MSCs and determined their multi-potent abilities by differentiating into osteoblasts, chondroblasts and adipocytes (Figure 1A-D). Qualified MSCs were then transfected with Ad-HGF and compounded with fibrin glue. Pseudopods of GFP-expressing MSCs in the fibrin glue composite could be clearly observed under microscope, suggesting the healthy state of these compounded cells (Figure 1E). The MSCs extended on the surface of fibrin glue composite with normal morphology as shown by SEM, while internal cells mainly displayed a sphere phenotype with abundant surface villi commonly seen on healthy cells (Figure 1F). These results suggest that fibrin glue-compounded cells can survive and remain healthy.

### Fibrin glue-compounded transgenic MSCs showed similar biological activities as uncompounded transgenic MSCs in short-term *in vitro* culture

**Figure 2:**
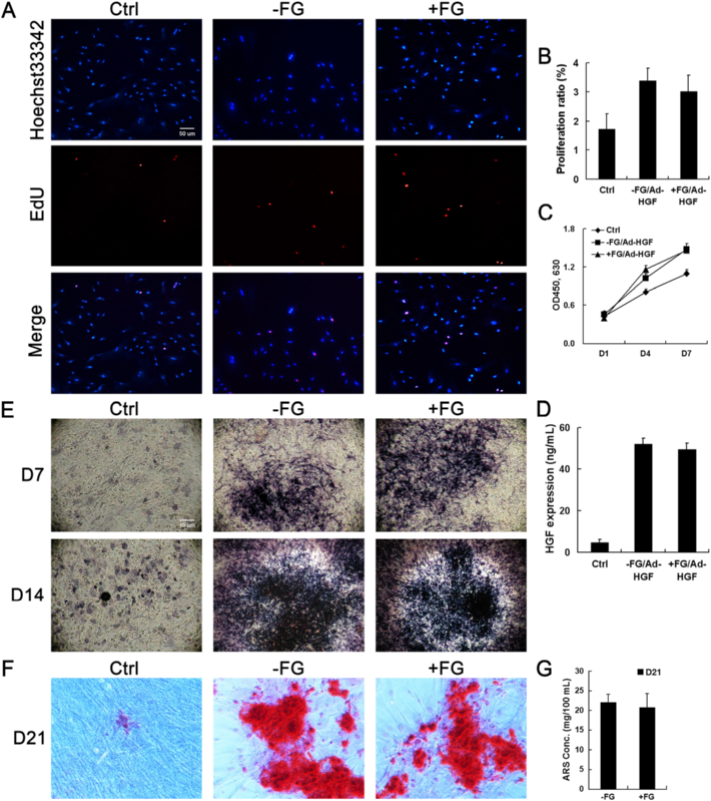
**Proliferation, HGF expression and osteoblast differentiation of MSCs compounded with fibrin glue.** Cell proliferation and viability were assayed using EdU incorporation **(A, B)** and CCK-8 method **(C)**. HGF expression was assayed using ELISA **(D)**. Osteoblast differentiation of MSCs compounded with or without fibrin glue was assayed using ALP staining **(E)** and ARS staining **(F, G)** after treatment with osteogenic culture medium.

To assess the effects of MSCs compounded with fibrin glue, we evaluated the proliferation and osteogenic differentiation *in vitro*. After dissolving the fibrin glue component of the MSC-fibrin glue complex one week later, the biological activity of compounded MSCs was determined and compared with uncompounded MSCs. The results of EdU incorporation and WST-8 assays showed that MSCs from the MSC-fibrin glue complex have a high proliferation rate similar as uncompounded MSCs resulting from the expression of HGF transgene (Figure 2A-C). The expression level of secreted HGF from transgenic MSCs was assayed using ELISA at 24 h after MSCs released from fibrin glue. The results showed that transgenic MSCs expressed similar level of secreted HGF no matter ever compounded with or without fibrin glue (Figure 2D). Moreover, MSCs from fibrin glue composite exhibited similar osteogenic differentiation ability as uncompounded MSCs, which was showed by ALP expression (Figure 2E) and alizarin red (ARS) staining (Figure 2F, G). The above results suggested that short-term compounding with fibrin glue will not change the biological activities of MSCs in osteogenic differentiation and responsive sensitivity to HGF stimulation.

### Fibrin glue composite improved the therapeutic effects of HGF-transgenic MSCs on ANFH

**Figure 3:**
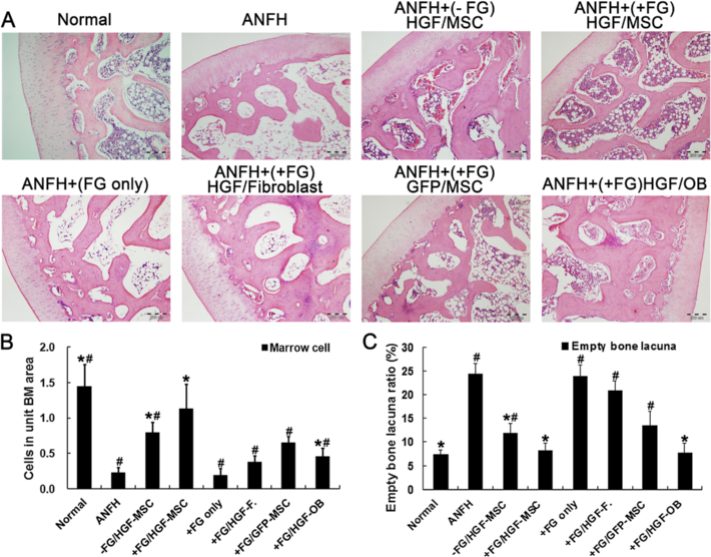
**Histopathological assessment of treatment efficacy with MSCs for hormone-induced ANFH.** Eigth weeks after MSC transplantation, HE staining results of histological examination **(A)** were semi-quantified for hematopoietic tissue in the medullary cavity **(B)** and empty lacunae in the trabeculae **(C)**. n = 15 or 30/group (3 sections/animal, 5 or 10 animals/group). *, *P* < 0.05 versus the ANFH group. #, *P* < 0.05 versus the MSCs + FG group.

After the ANFH model was established, the animals were given MSCs or MSCs + FG treatment. Similar as previous report, there is no significant difference could be observed between fibrin glue-compounded and uncompounded groups while obvious and similar therapeutic effects were observed in both treatment groups within 4 weeks [21]. However, the MSCs + FG group exhibited more significant improvement in recovery from ANFH when the observation period was extended to 8 weeks. Results of HE staining indicated that hematopoietic cells in the MSCs + FG group increased more significantly than that of the MSCs group and the number of empty lacuna was dramatically decreased in the MSCs + FG group compared with the MSCs group (*P* < 0.05) (Figure 3). The above results suggested better angiogenesis and formation of osteogenic microenvironment in MSCs + FG group during tissue repairing.

**Figure 4:**
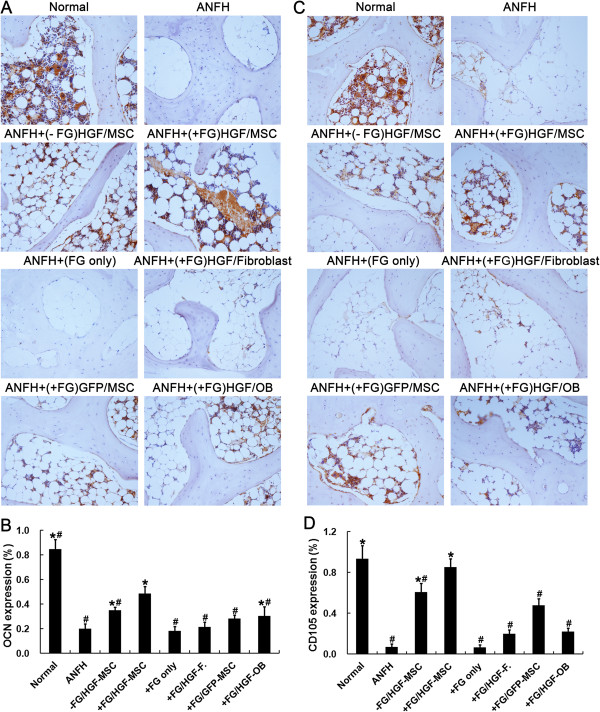
**Immunohistochemical detection and semi-quantification of *in vivo *OCN and CD105 expression.** OCN **(A, B)** and CD105 **(C, D)** expression were assayed following induction of hormone-induced ANFH and 8 weeks after treatment with transplantation of MSCs. n = 15 or 30/group (3 sections/animal, 5 or 10 animals/group). *, *P* < 0.05 versus the ANFH group. #, *P* < 0.05 versus the MSCs + FG group.

As a specific marker of bone formation during bone turnover, OCN could be used to characterize the initial osteogenic differentiation of MSCs [24,25]. In addition, OCN was mainly expressed in perivascular vessels and part of osteocytes in normal tissues. In ANFH model, expression of OCN was almost lost completely. Such loss could be rescued by transplantation of Ad-HGF transfected MSCs regardless of the presence of fibrin glue in short-term treatment. However, the significant differences only could be clearly observed at the end of 8 weeks post treatment, OCN levels in the MSCs + FG group was significantly higher than that in the MSCs group (*P* < 0.05) (Figure 4A, B). As expected, transplantation of HGF transgenic MSCs restored the vascularization of the femoral head indicated by the expression of CD105, the marker of newly-formed blood vessels [26,27]. Notably, the level of CD105 in the MSCs + FG group was also higher than that in the MSCs group (*P* < 0.05) at 8 weeks after MSC transplantation (Figure 4C, D). These results suggested that the duration of the MSC activity and the secretion of HGF were longer in the MSCs + FG group than that in the uncompounded MSCs group.

Significant improvement of ANFH defects compared with the FG group and the fibroblasts + FG group (*P* < 0.05) further demonstrated the effect of MSCs in the recovery of ANFH. Even being treated with osteoblasts combined with fibrin glue (the OB + FG group), the alleviation of ANFH symptom was also not equivalent as that in the MSCs + FG group which may be owing to the lack of vascularization activity by osteoblasts (Figures 3, 4).

### Sustaining expression of HGF and activation of signaling pathways contributed to the therapeutic effects

**Figure 5:**
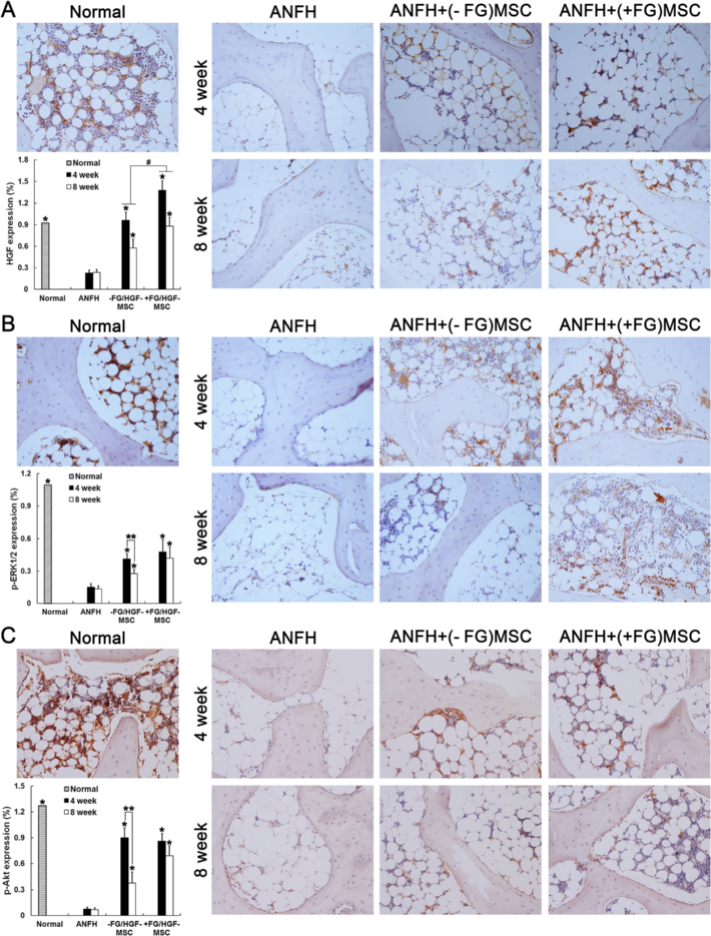
**Immunohistochemical detection and semi-quantification of *in vivo *HGF expression, p-ERK1/2 and p-Akt.** HGF expression **(A)**, phosphorylation of ERK1/2 (p-ERK1/2) **(B)** and Akt (p-Akt) **(C)** were assayed at 4 and 8 weeks after treatment of hormone-induced ANFH with transplantation of MSCs. n = 15 or 30/group (3 sections/animal, 5 or 10 animals/group). *, *P* < 0.05 versus the ANFH group; #, *P* < 0.05 versus between the MSCs group and the MSCs + FG group **(A)**; **, *P* < 0.05 versus between 4 and 8 weeks **(B, C)**.

To explore underlying mechanism, we detected HGF and downstream signaling molecules in the local regions. Consistently, the local expression of HGF was already higher in the MSCs + FG group than in the MSCs group (*P* < 0.05) by the end of 4 weeks post treatment. This situation of higher expression of HGF in MSCs + FG group was maintained until the end of 8 weeks post treatment (Figure 5), which was consistent with better therapeutic effects achieved in the MSCs + FG group. ERK- and Akt-mediated signaling pathways are downstream of HGF stimulation [28] and their crosstalk contribute the fine-tune regulation of osteogenesis. Our previous results showed that dosage variation of local HGF levels could significantly affect the activation of the downstream ERK and Akt signaling pathways, and consequently regulate the proliferation and differentiation of MSCs as well as MSC-mediated osteogenic differentiation during the treatment of ANFH [22]. Therefore, the activation levels of ERK and Akt signaling pathways were compared between the MSCs group and the MSCs + FG group. IHC staining showed similar level of ERK1/2 signaling activation between the two transgenic MSC-transplanted groups by the end of 4 weeks post treatment. The difference appeared between 4 to 8 weeks. In the MSCs group, the level of ERK1/2 activation fell significantly compared with that by the end of 4 weeks (*P* < 0.05). However, sustained activation of ERK1/2 was observed in the MSCs + FG group at 4 weeks and 8 weeks post treatment. As expected, the activation level of Akt pathway was decreased in the MSCs group significantly at 8 weeks compared with 4 weeks, while the level of Akt activation was maintained in the MSCs + FG group. In short, although HGF-secreting MSCs in the MSCs group contributed significantly to the recovery of local ANFH lesion, fibrin glue-compounded transgenic MSCs could maintain longer survival period of MSCs and result in prolonged HGF secretion time which provide more sustainable and effective therapeutic effects in treatment of ANFH.

## Discussion

In this study, the biological characteristics of MSCs after compounded with medical fibrin glue were compared with that of uncompounded MSCs *in vitro* and *in vivo*. Our results demonstrated that Ad-HGF-transfected MSCs compounded with fibrin glue not only prevented the loss of MSCs after the long-term (about 8 weeks) *in vivo* treatment in the femoral head but also allowed the prolonged and gradual release of HGF expressed by transgenic MSCs to improve the proliferation and osteogenesis of MSCs which contributed the ANFH therapy *in vivo*.

The characteristics and clinical usage of fibrin glue have been widely investigated and reviewed [29]. Although fibrin glue has been used as a cell delivery system, its effect on the activity of transgenic MSCs has not been clearly elucidated. We found that fibrin glue compounding could maintain the healthy state of MSCs as uncompounded MSCs which was supported by live cell imaging and SEM observation showing the imbedded MSCs with abundant surface villi of sphere MSCs. *In vitro* assays showed that MSCs released from MSC-fibrin glue complex exhibited similar levels of HGF secretion, proliferation and osteogenic differentiation compared with uncompounded MSCs. Thus, fibrin glue compounding, as a physical cell delivery system, could completely maintain the full biological characteristics of MSCs.

Short-term *in vivo* assays showed that fibrin glue compounding or non-compounding displayed no significant differences in therapeutic efficacy by the end of 4-week treatment. However, the differential therapeutic effects were apparent that the MSCs + FG group exhibited more significant advantages than those uncompounded groups when the treatment period was extended to 8 weeks. The better therapeutic capacity of MSCs + FG group was demonstrated by more hematopoietic medullary cells and less empty lacunae formation as showed by H&E staining. And elevated expression of OCN and CD105 indicated the improvement of osteogenesis and haemopoiesis. It was supposed that the better performance of MSCs + FG group resulted from the sustainable viability and elongated HGF secretion of MSCs in the local lesions was due to fibrin glue compounding, which significantly improved the efficiency of treatment with MSCs. Effects of longer survival time *in vivo* could be observed as early as 4 weeks and last to 8 weeks which assured the stable and longer secretion of HGF by MSCs to improve the bone marrow microenvironment and promote proliferation and differentiation of themselves as well as other cells in the femoral head, thereby promoting amelioration of ANFH.

Our previous results demonstrated HGF-dosage-dependent regulation of the downstream signaling pathway mediated by c-Met, the HGF receptor, to regulate the cell fate of MSCs *in vitro* and *in vivo*[22]. Local high expression of HGF (about 100 nM) which appeared within 2 weeks after transplantation of HGF-transgenic MSCs preferred to activate ERK signaling and in turn promote cell proliferation, In contrast, local lower expression of HGF, about 20 nM appearing after 2 weeks after MSC transplantation tended to activate Akt signaling which improved the osteogenic differentiation of MSCs [22]. In this study, significant decrease of activated ERK and Akt signaling could be observed *in vivo* in uncompounded MSC-transplanted ANFH animal models. Loss of ERK caused by the quick decrease in the level of HGF just like what we observed in previous studies [22]. The significant downregulation of Akt activation further indicated the relatively quick loss of the osteogenic capacity of MSCs in the local region. However, the higher expression levels of ERK and Akt in the MSCs + FG group were maintained for a relatively longer period than the MSCs group. Fibrin glue-compounded MSCs showed dramatically-extended survivability and prolonged secretion of HGF which promoted the proliferation and the following osteogenic differentiation of MSCs *in vivo* than uncompound MSCs. Thus more MSCs with enough time to fully differentiate into osteoblasts provided sufficient therapeutic cells for ANFH lesion repairing. These results proposed the important role of fibrin glue in supporting the HGF-secreting MSCs in the local lesion region for sustainable therapeutic goal.

## Conclusion

Using MSCs compounded with medical fibrin glue provides a promising new regime which could greatly improve the efficacy of clinical therapy of ANFH.

## Abbreviations

MSCs: Mesenchymal stem cells; ANFH: Avascular necrosis of the femoral head; HGF: Hepatocyte growth factor; Ad-HGF: Adenoviral vector-based hepatocyte growth factor; SEM: Scanning electron microscopy; HE: Hematoxylin-eosin; OCN: Osteocalcin; ARS: Alizarin red.

## Competing interests

The authors declare that they have no competing interests.

## Authors’ contributions

QW conceived and designed research, acquired data, analyzed and interpreted data and results, performed statistical analysis, and drafted the manuscript. CZ prepared samples, analyzed data, and reviewed and provided feedback during the development of the manuscript. WL provided conceptual advice, analyzed data, and participated in discussion of results. MZ conducted the animal experiments and prepared samples. LM contributed to the scientific direction, experimental approach, and interpretation of results and revision of the manuscript. All authors read and approved the final manuscript.
